# Policy actors as data integrators: mixing data in tobacco control evaluation and assessment

**DOI:** 10.1017/S1744133126100619

**Published:** 2026-07-27

**Authors:** Claire L. Ma, Holly Jarman, Praneetha Vissapragada, Jamie Hartmann-Boyce

**Affiliations:** 1 Health Management and Policy, University of Michigan School of Public Health, USA; 2 Health Policy and Promotion, University of Massachusetts Amherst School of Public Health & Health Sciences, USA

**Keywords:** data integration, evidence-based policy, public health, qualitative methods, tobacco control

## Abstract

Tobacco control policies are complex with the potential for significant health effects. The public health actors and regulators who design, influence, and implement these policies place a high value on the evidence base that underpins them, engaging in scientific and applied debates about how to evaluate the success of existing policies and justify new actions. To investigate the construction of this evidence base, we conducted interviews with 40 individuals involved in formulating, advising, and implementing flavour restrictions for tobacco products. Our interviews inform case studies of six jurisdictions in three US states, Canada, the UK, and the Netherlands. We find that tobacco policy stakeholders, which include regulators and public health personnel, play a vital role as data integrators in the construction of this evidence base: they bring together disparate types and sources of information to create synthetic evaluations of tobacco control policies that incorporate the complexity of the policy environment and differential impacts on population subgroups. Significantly, data integration is not easy to replicate or automate; should these positions be lost due to funding cuts as political priorities shift in the US and globally, the capacity they add to our policymaking and research systems will be difficult to restore.

## 1. Introduction

Policies targeting tobacco products are complex, detailed, contested, and often short-lived (Ling et al., 2022). They vary considerably by jurisdiction and by product category, while rapidly changing markets for products contribute to an unstable environment for implementing restrictions. While scientific evidence of the health effects of tobacco products such as cigarettes and chewing tobacco is well-established, the evidence base for the health effects of newer products is often incomplete or inconclusive (CDC, 2024b; McNeill et al., 2022; NASEM 2018; Cohen and Cook, 2025; Travis et al., 2022, 2025; WHO, 2025). Data on which tobacco products are used, how, and by whom is vital for successful policy making and implementation; however, this data is difficult to obtain outside of real-world observation. As a result, tobacco control regulators and public health actors must constantly refine policies through an iterative process to contend with commercial and corporate interests such as evolving industry strategies to evade regulations and influence evidence, threats of litigation, and new forms of advertising, promotion and distribution (Fooks et al., 2013; Gilmore et al., 2023; Kickbusch, 2012). This conflictual environment leads tobacco policy stakeholders – which refers to tobacco control regulators and public health advocates working on tobacco control in public health agencies and organisations at the local, state, and national levels – to place a high value on the collection, synthesis, use, and documentation of evidence in decision-making about tobacco and nicotine.^1^

Our study demonstrates how, to navigate these challenges, tobacco policy stakeholders working to design and implement tobacco control policies act as flexible data integrators, bringing together multiple types of secondary and primary data to evaluate the success or failure of existing tobacco control policies and plan new ones. Data integration is a process used in qualitative, mixed method, and multi-method research^2^ in which different types of qualitative and quantitative data are combined to draw conclusions about a complex topic (Fielding, 2012). For instance, data integration in qualitative and multi-method research, also called QMMR, is an umbrella term in social science methodology that refers to combining and triangulating among various qualitative approaches such as interviews, document and policy review, and in-depth case studies (Büthe et al., 2015). Data integration is difficult to automate and can take place in many ways, including by designing studies that ask integrative questions, by combining datasets together through novel approaches, synthesising findings across distinct types of data during analysis, displaying connections between several types of data including unstructured data, and building narratives that align findings across different information sources. Importantly, data integration can be done with cross-sectional and longitudinal data offering flexibility to build evidence on quickly shifting markets. Tobacco policy stakeholders, as we document below, emphasise data integration as a core part of their policy practice.

As political priorities shift in the US and globally, public health actors face significant funding cuts that threaten their integral role in the health policy process. The US Food and Drug Administration (FDA) has recently begun to explore the use of AI tools in tobacco control, though anecdotal evidence suggests that these tools are not yet equipped to replace the human capital required to analyse and interpret diverse, unstructured, and potentially contested information (FDA, 2025). In this study, we identify five ways in which policy actors working in the subfield of tobacco control contribute to governance capacity through data integration. They mix data about the tobacco policy context, the market environment, and implementation to inform (1) assumptions, (2) temporality, and (3) comparison in evaluations and to improve (4) data quality and the (5) relevance of the work to the public and communities.

A combined understanding of existing research findings and the policy environment informs assumptions about problem definition, measurement selection, and the logic of causal mechanisms. Actors use interviews, surveys, and legal assessment to provide a richer understanding of temporality, e.g. the stages of implementation and when costs and benefits accrue. They seek to understand how policies are implemented in other jurisdictions, comparing these experiences to separate out global trends from national or local effects. They triangulate between data sources, including unstructured and non-public information, to gain insights into data quality and limitations. Lastly, they aim to improve the relevance of policy evaluations to populations affected by tobacco use, by collecting data with and about underrepresented groups and evolving market structures.

In the following sections, we elaborate on the role of evidence in tobacco control policymaking, the challenges of evaluating tobacco flavour policies, and the limitations of relying primarily on quantitative and statistical methods in evaluation. We then describe our methods to understand how tobacco control policymakers approach data in real-world cases. The remaining sections present our findings, elaborate on each of the five categories, and discuss the importance of methodological considerations when designing and conducting evaluations in tobacco control.

## 2. Constructing evidence on flavoured tobacco products

Tobacco control is a complex, high-conflict policy domain. Policies aimed at tobacco products are salient for individual users, potential users, communities, schools, retailers, restaurants and hotels, product manufacturers, health providers, and many other groups. The potential harms from tobacco products and potential profits from their sale are enormous. Debates about tobacco control touch on moral judgements about healthy behaviours and politically constructed views about key subgroups in the population, including children and young adults, older adults, and marginalised racial and ethnic minorities. Meanwhile, the tobacco industry protects its products and profits through political lobbying, litigation, and market diversification (Hiscock et al., 2023; Jacobson and Wasserman, 1999; Levy et al., 2023). As a result, almost all tobacco policies are contested, while many are heavily challenged across political, bureaucratic, and legal processes. These debates combine questions of values as well as disputes over evidence, informing both whose interests should be prioritised as well as justifications for why certain interests should be prioritised in laws and policies.

The complexity, contentiousness, and high-stakes nature of tobacco control lead policy actors across the domain to care a great deal about the evidence base for new policy decisions. Because products and the regulations that control them are constantly evolving, and because each new policy implemented is likely to attract legal challenges, many policy actors in tobacco control are involved in a continuous cycle of data and evidence generation, the evaluation of existing policies, and the impact assessment of new ones.

Although combustible tobacco products remain by and far the largest source of revenue for tobacco companies, the tobacco and nicotine product market has diversified significantly in recent years with the introduction of products including e-cigarettes and vapes that contain tobacco and/or nicotine, ‘heated’ tobacco products, and oral nicotine pouches (CDC, 2024a). Evidence about the harms of smoking older products like cigarettes and cigars is very clear, with a strong scientific consensus that they are extremely hazardous to health (CDC, 2024b; WHO, 2025). However, evidence on the health risks of newer products is still emerging, with opinions among scientific experts more divided when it relates to how evidence should be translated into policy (McNeill et al., 2022; NASEM, 2018; Cohen and Cook, 2025; Travis et al., 2022, 2025).

Manufacturers of tobacco and nicotine products use added flavours and related sensory cues such as smell or cooling/numbing sensations,^3^ packaging, and descriptors to attract users (Cohen et al., 2021; Pullicin et al., 2020; Talhout and Leventhal, 2026). Research shows that adding flavours to combustible tobacco products can worsen their damaging effects. For instance, candy and sweet flavours in little cigars make these products more attractive to children (Audrain-McGovern et al., 2025). Clove cigarettes provide numbing that counteracts some of the negative sensations experienced when starting to smoke, easing the transition to becoming a smoker (Cohen et al., 2024). Users of menthol cigarettes inhale more deeply than people using regular cigarettes, with even worse health consequences (Wickham, 2020).

For newer tobacco and nicotine products on the market, such as non-combustible and synthetic nicotine products, there is increasing evidence that flavours can attract new users who have otherwise never smoked combustible tobacco.^4^ Sweet and fruity flavours in e-cigarettes appeal to children and young adults, but research has also shown that older adult users of e-cigarettes find flavours appealing (Leventhal et al., 2020; Livingstone-Banks et al., 2025). Flavours therefore play a potential role in attracting smokers away from cigarettes and cigars and towards lower-risk products (Lindson et al., 2025; Livingstone-Banks et al., 2025).

In recent years, governments have become increasingly interested in addressing the appeal of flavoured tobacco and nicotine products, especially their potential draw for first-time users among children and young people. Recent research suggests that while some countries, including the US, Canada, the 28 member states of the EU, and a handful of other countries, have comprehensive or partial bans on flavours in tobacco and nicotine products, the majority of countries still do not have any de jure restrictions (Erinoso et al., 2021; Ma et al., 2026; Campaign for Tobacco-Free Kids, 2026; Whitacre et al., 2025).^5^ Established restrictions cover flavours in older tobacco products where the evidentiary basis is well-established, such as the US federal ban in 2009 that prohibited all non-tobacco flavours in cigarettes except for menthol. In some places, policymakers are expanding these restrictions through new laws and regulations that cover newer flavoured products. The result is considerable policy variation in the US and globally. In each case, restrictions on the sale of flavoured products sit alongside complex regulations about product manufacturing, importation, purchasing, retail licensing, and rules about marketing and product promotion.

In addition to emerging science on new products and the diversity of regulatory approaches taken by governments, legal threats from the tobacco industry add to complex debates about evidence. Big tobacco firms are famously litigious, especially in the US, but increasingly in many other jurisdictions, and such legal threats influence the policymaking strategy of tobacco control advocates and can shape the scope and design of regulations (Peck et al., 2022). The tobacco industry has lobbied aggressively against efforts to expand flavour bans and threatened lawsuits against restrictions limiting public health outcomes tied to stricter regulation (Derthick, 2011; Silva et al., 2019). At the same time, the tobacco industry has circumvented existing regulation by redesigning products. In response to this policy environment, flavour restrictions have become iterative and incremental. The recent introduction of ‘non-menthol’ menthol cigarettes into the tobacco product market, for instance, motivated public health officials to identify limits of their existing flavour laws and reconsider legal definitions for flavour restrictions (Hiscock et al., 2020).

When it comes to evaluating the effects of existing and potential policies, variation in jurisdictional authority and capacity, population health, tobacco product landscape, and market conditions make generalisable impact assessment more difficult. While quantitative evidence is critical for tobacco policy stakeholders to measure and evaluate these policies, qualitative investigations provide contextual information that helps researchers and policymakers to determine the scope and generalisability of any evaluation and the value of future policy actions. Evaluation efforts often occur in environments in which structured data may not yet be publicly available. In these cases, qualitative and mixed methods approaches can fill evidentiary gaps, provide relevant details about the specificity or generalisability of findings, and contextualise other sources of information.

Evidence-based policy often prioritises cumulative empirical evidence from randomised controlled trials, including both quantitative and qualitative data, that can help policymakers identify factors contributing to policy adoption, effective design, and desirable outcomes (Brownson et al., 2009). The approach first emerged during the mid-20th century to assess medical interventions and later expanded into social policy in employment, education, and welfare reform to support more cost-effective and accountable use of public and private resources (Baron, 2018; Head, 2016). However, evidence-based policy cannot be decoupled from its context; policies and policy change are also shaped by ideas and ideologies, as well as the institutions in which policy actors interact (Andersen and Breidahl, 2021; Béland, 2005, 2009; Prinja, 2010).

Research at the intersection of political science and public health demonstrates the political, constructed nature of evidence and its strategic use by public health actors in the policy process (Clavier et al., 2022; Fafard et al., 2022). In the context of public health, prior work on the subject has shown how public health actors use evidence to make arguments for policy, framing evidence in strategic ways and establishing relationships with other interest groups (Clavier et al., 2022). Emotional appeals, political pragmatism, and strategic communication also affect the extent to which evidence is translated into policy (Cairney and Oliver, 2017). Researchers mix qualitative and quantitative data to study complex cases in ways that allow them to adapt when case conditions change (Creswell et al., 2011). Integrating qualitative data provides opportunities for practitioners, such as public health officials and regulators, to engage in the evaluation process (Poth et al., 2024; Spector and Pinto 2017).

It is in this context that we consider the specific approaches to data integration used by stakeholders working in tobacco control, alongside more traditional quantitative evaluation tools, to improve tobacco control policy assessments. The following sections lay out our methods for investigating this phenomenon, followed by a detailed discussion of our findings.

## 3. Methods

Our research team is part of a US federally funded Tobacco Center of Regulatory Science (TCORS) that involves research collaboration across multiple higher education institutions in the US and abroad (Wipfli et al., 2017). The centre partners with the US FDA and National Institutes of Health (NIH), as well as a nationwide committee of external tobacco control experts who inform our research. Federal partners and the expert committee provided feedback on the initial research design that underpins our findings.

For this project, we relied on case studies that included a number of elite interviews with people from each of the case jurisdictions to understand the data integration practices of tobacco policy stakeholders, especially those involved in tobacco policy evaluation and assessment. Elite interviews focus on small populations of individuals with exclusive information about a topic that is not easily substitutable, such as policy officials or groups of professionals engaged in a specific policy area (Mosley, 2013). Jurisdictions were selected based on input from the centre’s federal partners about the relevance of the cases for US policymakers, primarily if they had recent experience passing and implementing tobacco flavour restrictions. We conducted preliminary research on potential cases in order to purposefully select six cases of comparably high-income democratic countries with de jure tobacco and nicotine flavour policies at the national level but that included broad variation in the type of flavour regulations in place.

To investigate specific ways in which data integration is used in real-world assessments of flavour regulations, we conducted interviews with 40 public health experts and regulators involved in tobacco/nicotine product policy and evaluation in three US states, Canada, the UK, and the Netherlands. Table 1 includes the summary of the policy contexts of our cases, along with information about our interviewees.

We opted for an elite interviewing model because the universe of suitable interviewees is relatively small. Interviewees were drawn from public health, research, and regulatory organisations at different levels of government; our interviewees are anonymised in order to protect the confidentiality of our participants.^6^ Our interviewees were purposefully sampled based upon their role and expertise in tobacco control and include individuals involved in advisory roles, policymaking, programme evaluation, and retail inspection. We reached out by email to a total of 75 individuals, of which 40 agreed to be interviewed and included in the study under conditions of anonymity and confidentiality. The semi-structured interviews followed a standard protocol and included questions about data use, data sources, and evaluation efforts. Two researchers from our team conducted each interview. Interviews were only attended by the participants and researchers. The interviews lasted approximately 1 hour each and were recorded and transcribed using AI software (Zoom), corroborated by interviewer notes. Interviewees were interviewed only once, and the researchers wrote reflective memos after each interview had concluded. It is not typical to employ member checking in elite, semi-structured interviews.

We subjected the text corpus to thematic analysis (Saldaña, 2015). Two researchers inductively and manually coded the text corpus using NVivo 15. We conducted three rounds of open coding, acting independently for each round before discussing the results to achieve consensus. The coded interview transcripts were then analysed to identify common themes about data use in tobacco/nicotine regulation and evaluation. In addition to understanding how our interviewees use or approach data integration in their work, we also identified ways in which the qualitative interviews and case studies contributed to our assessment of flavour restrictions across the six jurisdictions.

As researchers in the tobacco control space, qualitative methodologists, and active participants in translating evidence to policy, we acknowledge that our background informed the development of the research questions and provided familiarity with the policy context discussed by our participants. CM, HJ, and JHB have PhD qualifications, PV is a doctoral student. Three researchers are female and one is non-binary. All have received training in the relevant qualitative methods. More information about our methods, including participant recruitment, interview protocol questions, and a qualitative methods checklist, can be found in Supplemental Materials A, B, and C.

The research design was reviewed by the IRB at the University of Michigan and classified as unregulated. All protocols conform with standard practice for the General Data Protection Regulation (GDPR).^7^

## 4. Findings

Policy evaluation and assessment involve multiple components including defining the problem, understanding timing and policy history, determining variation and generalisability, assessing data availability and limitations, and applicability or representativeness of the outcomes (Kroneman and van der Zee, 1997; Skivington et al., 2021). In analysing our interviews, we inductively identified and grouped interviewee comments on data use and analysis into five key categories to describe how tobacco policy stakeholders approach data in evaluating existing policies, revising them, and creating new ones. Data integration formed a core component of policy practice among these actors, informing (1) assumptions made in evaluations about the problems to be addressed and the policy environment, the (2) temporality of assessments, such as the time periods to be covered by analyses, and (3) comparison between policies, systems and patterns of use in different jurisdictions. Data were mixed in order to improve (4) data quality by providing needed context and potential explanations for patterns and to improve the (5) relevance of the work to those affected by use of tobacco products. This data includes information across the tobacco policy landscape from understanding the landscape of the market and existing and emerging products, existing policies, and limitations in implementing regulations in practice.

We summarise the five categories of data integration with illustrative quotes from our interviews in Table 2.

Figure 1 summarises the categories and ways in which actors perform data integration in relation to various assessment components. In this section, we describe how tobacco/nicotine regulators approach data integration and why these approaches are helpful for assessing tobacco control policies. We discuss each of the five categories in turn and provide examples from our interviews.^8^

### 4.1. Assumptions

In evaluation, correct assumptions about the logic of causal relationships are important because they go on to inform which variables are selected, how they are measured, and the analysis itself (Mertens, 2016). In environments in which data are not yet public or available in structured form, interviewees mixed data to identify and address the assumptions held by policymakers and the public about tobacco products and policies. Interviewees anticipated a need to provide evidence-based narratives that addressed faulty assumptions held by political decisionmakers, who often differed in their policy goals and priorities, experiences and understandings of products, and populations or communities of interest.

Interviewees described efforts to improve their assumptions by developing an understanding of the policy context, e.g., by understanding the effects of ‘flanking’ or proximate policies that can interact with the policies under study, or describing the institutional, market, and media contexts in which regulators operate. ‘Flanking’ policies interact with tobacco flavour restrictions and influence the contexts in which flavour restrictions are implemented, including marketing and advertising laws, age restrictions, and other sale limitations such as policies on the proximity of tobacco retailers to schools.

Among interviewees, understanding assumptions was seen as critical for making relevant conclusions. Doing otherwise risked reaching the wrong conclusions, leading to policy recommendations that were inappropriate or likely to fail. When seeking to inform policymaking, interviewees describe a loop of activities, where they are ‘using data, whether from [my jurisdiction or others], and trying to help educate policymakers and stakeholders to pass some kind of tobacco regulation … and also trying to do evaluation of those policies’ (#26-US, public health agency, local). Actors translate data in ways that ‘tell a story, not just showing them the data.’ This occurs both during policy formulation and during implementation, when they ‘continue to show the data that it’s working.’ This follow-up during the implementation phase was seen as essential due to the high conflict surrounding tobacco control policies. In these contexts, ‘the biggest piece is evaluation of the actual programme so that the policy can withstand any sort of scrutiny or overturn’ (#14-US, non-profit, state).

Engaging with evaluation from an early stage and continuing it through ongoing implementation means actors are ‘able to go back to the data and evidence that points to the effectiveness of [prior policy] campaigns’ and weave this into narratives that help policymakers consider how future policies might play out (#25-US, public health agency, state). This translational loop is seen as an important counter to the ‘academic narrative of we’re giving reams and reams of data and evidence, and then it will just translate. It just doesn’t work like that.’ Actions that bring understandings of data and values together are required (#17-UK, non-profit, state).

Interviewees described various ways in which they mixed data when engaging in these translational activities. Often, they would match survey data asking about product use to retailer or sales data, from which they could craft narratives that more closely defined the problem. Several interviewees mentioned conducting GIS mapping to explore retailer density, sometimes combined with an analysis of populations in neighbourhoods where certain products are being sold (#6-US, public health agency, local; #10-US, public health agency, state). From these ‘heat map’ analyses, actors could determine that ‘certain flavour use was happening in certain areas of [the jurisdiction] and there were more minors that would purchase products in a certain area of [the jurisdiction]’ (#23-US, public health agency, local). Mapping was an important part of problem definition because while some forms of data can indicate the scale of product use, actors also needed to answer frequent questions from policymakers such as ‘where are young people getting these tobacco products from? Because that affects what solution we think will be promising’ (#40-US, public health agency, local).

Tobacco policy stakeholders crafted narratives by mixing insights from published research and qualitative reports on emerging products and use trends – from journalists, schools, doctors and others who would ‘ring the alarm bell’ – with lab data on product effectiveness, retail surveys, and market and use data (#23-US, public health agency, local). Mixing information from actors on the ground with media tracking and evaluation of cessation data was seen as providing a much more robust picture of use in the context of an evolving market for products (#28-US, public health agency, state). Interviewees described the utility of systematic evidence reviews alongside ‘alarm bell’ insights when seeking to inform policymaking:

‘I’m part of a unit that is doing systematic evidence reviews and scoping reviews. It’s our job to funnel that into this conversation alongside these more qualitative conversations that I’m having. Hopefully, we’re giving the decisionmakers something that is structured on the evidence as it exists’

(#24-Canada, research, state).

Mixing data gave actors a window into complex questions of policy implementation and compliance. For instance, ‘when you get surveillance data showing that either vaping has not changed or not decreased [despite a product ban], you can link it to the fact that it’s noncompliant [behaviour]. It’s not the regulation itself’ (#33-Canada, non-profit, state) Tobacco control actors might know from systematic reviews ‘how effective e-cigarettes are’ at helping people to quit smoking. But by triangulating reviews with survey data they can also determine ‘for the people we are trying to reach, if they’ve used alternatives [other than e-cigarettes] and it hasn’t worked for them’ (#39-Canada, public health agency, national).

Some interviewees talked about how they use methods from epidemiology and the social sciences to integrate understandings of how tobacco product use might interact with other potentially addictive or harmful products that are often in legal grey spaces, such as cannabis or psychedelics (#35-UK, research, national; #36-US, research, local). There was a perceived need to understand sales and use across multiple categories of products, even for a narrow policy, because restricting one product could cause people to switch to another that might be less desirable (#30-Netherlands, public health agency, national; #27-US, public health agency, state; #31-US, public health agency, state).

Interviewees emphasised that a range of policies interact with tobacco flavour restrictions and influence the environments in which flavour restrictions are implemented. Some of these policies, which we term ‘flanking policies,’ provide scaffolding for flavour restrictions and affect actors’ evaluation of the effectiveness of flavour policies. This was seen as an important limitation on policy evaluation (#34-UK, non-profit, state). Understanding which flanking policies were in place was seen by interviewees as essential to avoid drawing the wrong conclusions from evaluations.

Cessation support, limits on the age at which products could be sold, and tackling cross-border product flows were all cited as important flanking policies for flavour policy implementation:

‘What I would like to see is that the age limit for buying tobacco could be raised from 18 to 21, and to go to a generational ban, every year, one year above the age limit … But if there aren’t other measures to help people who are still smoking and they are addicted, they don’t care if they can buy it or not –they will find a way to get it’

(#30-Netherlands, public health agency, national).

‘If the Chinese [export of flavoured tobacco/nicotine products] were shrunk down a bit, if the manufacturing capacity were reduced, it would probably pop up somewhere else. …So [you need] the use of an international instrument to negotiate this … to minimise the supply in legal ways’

(#20-Canada, non-profit, national).

When trying to understand problems affecting certain population subgroups, it was also seen as important to look beyond tobacco, using mixed qualitative and quantitative approaches, to see what other products groups might be using and seek to understand those policy areas (#35-UK, research, national).

Beyond flanking policies, interviewees discussed how the political and institutional contexts of different jurisdictions affect how regulators define the problem of flavoured tobacco and the tools available to address the problem as defined:

‘Defining the problem requires that you look at the political and institutional context, like how the powers are divided between [National] and [Subnational] governments and what responsibilities are given to [Health Officials] to apply the [core tobacco legislation], which is quite different from the [core drugs legislation] and the [core consumer protection legislation]. Those considerations affect how we address a problem – how we look at evidence, the threshold that is needed for the [Health Official] to take action, and what the courts expect in terms of evidence’

(#37-Canada, public health agency, national).

Interviewees described the need to navigate these institutional differences when seeking to educate policymakers about the nature of tobacco control problems (#35-UK, research, national; #26-US, public health agency, local; #34-UK, non-profit, state; see also ‘comparison’ below).

### 4.2. Temporality

A second use of data integration by tobacco policy stakeholders is to provide information about the timing of policy implementation and enforcement, or temporality. This includes information about when the policy is ‘in force’ as a matter of law, in place as a matter of practice, and when the costs and benefits of policy accrue. Interviews, surveys, data dictionaries (and other qualitative assessments of data quality), and expert input were all used by interviewees to provide a richer understanding of temporality considerations. Interviewees recounted that qualitative data is especially important for identifying the stages of implementation that may not be reflected in official dates for enforcement (#27-US, public health agency, state).

When the aim is to evaluate the success of a single policy intervention, some interviewees shared notes of caution about how overlapping policies can impact conclusions. Sometimes policies have ‘all been introduced at the same time, and it’s very difficult to be able to say, this is the impact … I think it’s just not possible because of the way that tobacco policies are implemented and a lot of them have small but incremental impacts’ (#34-UK, non-profit, state).

These temporal considerations apply regardless of the level of government (#27-US, public health agency, state; #26-US, public health agency, local; #35-UK, research, state). When considering national policies from a global perspective, ‘you’ve actually got to look at the level of implementation [and not just the date of implementation] because most countries … they probably try and make sure most of the measures are enforced … But most countries don’t [enforce all policies before they ratify international agreements like the Framework Convention on Tobacco Control].’ International agreements set the expected standard for tobacco control policies, but they do nothing to guarantee implementation of laws that are in place (#34-UK, non-profit, state).

One reason that temporality matters a great deal in tobacco control policymaking is because product manufacturers frequently introduce new products and types of products onto the market. Interviewees noted that industry adapts rapidly to any new policy restrictions, sometimes making changes in their products to explicitly evade rules and regulations. New products and formulations of these products need to be evaluated and evidence of their likely health effects considered. This requires tobacco policy stakeholders to rapidly integrate data from emerging studies. Systematic reviews, particularly when they are frequently updated (sometimes known as ‘living reviews’) are highly valued, because ‘we need that kind of dynamic research, which is continually refreshed, to inform our evidence piece of our policies’ (#15-UK, public health agency, national).

### 4.3. Comparison

Interviewees also talked about making accurate comparisons across jurisdictions or settings. Comparison is often used in evaluation to understand variations in policy conditions and outcomes across cases. Comparisons support more accurate generalisations and help identify global trends versus national or local effects. Comparative and in-depth case study approaches are uniquely primed to address questions about the scope of study and the potentials, or limitations, for generalisability (Gerring, 2007; Ragin et al., 1998). Using comparison in case studies can range from controlled comparisons of two carefully selected cases to multiple case studies that provide information about a range of policy implementation contexts (Gupta, 2012).

In the context of policy evaluation, interviewees mentioned conducting comparative policy surveillance, soliciting expert opinions from their counterparts in other jurisdictions, and using existing summaries such as grey literature from advocacy groups, international surveys, and policy scoping reviews to evaluate the appropriateness of comparisons. Legal epidemiology, which involves the systematic tracking of public health laws and policies across different jurisdictions, was seen as a useful qualitative technique to generate relevant information for comparison. By providing details about legal contexts, legal epidemiology allows researchers to compare and contrast jurisdictions to identify the differences between policies that may affect public health (Ramanathan et al., 2017).

In tobacco control, studying ‘[variation across different jurisdictions] is a great opportunity for learning from different policies’ that can be applied to a home jurisdiction (#15-UK, public health agency, national). Interviewees mentioned that they frequently share information across borders about their policy experiences, which required knowledge of the differences between both political systems and policies. This was seen as a challenging task. Some interviewees pointed to the limitations of generalising to the country or larger jurisdiction, given differences between countries (#3-Netherlands, research, national) and subnational variation in policies (#37-Canada, public health agency, national). Others explicitly compared countries’ approaches:

‘When you look at flavours the way we’ve approached flavours, it’s different … So if we say that flavours help make the products more attractive, then the answer is, let’s not allow any flavours being added … characterising [flavours] does not fit anymore as the answer. When you define the problem as being a reduction of attractiveness, the proposed solution is different. That’s why, for tobacco products or cigarettes in particular, we went to having zero addition of any [characterising or not] flavours at all’

(#37-Canada, public health agency, national).

‘In [other jurisdictions], major groups in political life support a package of tobacco control measures. That’s not the case [in our jurisdiction]. I would say that [other jurisdictions are] better placed for such a measure than [our jurisdiction] right now’

(#30-Netherlands, public health agency, national).

Substantive epistemic networks have existed in tobacco control for a long time (Cairney et al., 2012). Interviewees discussed how they participate in networks of experts that seek to inform policy development in other places, e.g., to learn from past legal impediments such as falling afoul of policy wording that has already been legally contested and to utilise research resources efficiently. This participation includes leveraging multiple data types including comparative legal analysis, research on product specifications, and analysis of industry strategies (#4-US, public health agency, national, #35-UK, research, national, #30-Netherlands, public health agency, national, #39-Canada, public health agency, national). They sometimes allowed other jurisdictions to use data they had compiled, as ‘talking to countries who are looking to advance similar measures is helpful, and learning from their experiences as well, which is something that we kind of do informally’ (#39-Canada, public health agency, national).

Interviewees expressed the difficulties of struggling to synthesise large amounts of data from different jurisdictions while still facing considerable uncertainty when making policy decisions. A core problem is that ‘there’s not enough data in any given moment to conclude 100 percent we have to go right and not left. So there’s always this large amount of doubt … there’s so much research and there’s so much data. But still you are quite alone, and you have to reach these decisions on the basis of the best available data’ (#30-Netherlands, public health agency, national).

In this context, looking to other jurisdictions ‘where the industry really tries out new products’ was seen as valuable in terms of predicting what may come next, as ‘you see a lot of [product] promotions still that are not allowed in [my jurisdiction] that we see in [the neighbouring jurisdiction]’ (#3-Netherlands, research, national). Because supply chains do not necessarily respect borders, coordinating across jurisdictions was seen as a key part of controlling illicit product use (#38-Netherlands, public health agency, national). Having access to data on sales in other jurisdictions with different policies was also helpful in:

‘understanding trends in use and help frame the development of different policies … banning flavoured and pod based devices in [another jurisdiction] has led to a huge increase in popularity of disposable devices … I think monitoring sales closely and having that data being shared to policymakers [is useful], just so that people can be very nimble and quick in understanding where products are heading and identify potential loopholes’

(#24-Canada, research, state).

### 4.4. Data quality

In public health, understanding the quality of data is crucial for improving its interpretation and avoiding erroneous conclusions. Interviewees discussed using approaches such as qualitative interviews, surveys, and data notes to shed light on data quality. This included improving understanding of data limitations by talking to people who structure or use the data and thus providing information about data quality that is not yet publicly available. Interviewees sought to understand limitations such as a lack of completeness or representativeness in data and the timeliness of data collection and reporting, as well as to provide guidance on its use and misuse (Chen et al., 2014).

We heard from interviewees about gaps in the existing data and efforts to collect richer data from individuals and focus groups. As seen in our conversations about the timing of policy implementation and discussed in the section about temporality, interviewees involved in data analysis for their departments provided caveats about features and completeness of the data that influence how researchers should interpret publicly available data:

‘We’re collecting all this data. And all of this data goes into a repository. The repository was built – I don’t know – it’s probably older than I am. It probably was built back in the eighties. The thing is an ancient relic. The utility of it is terrible …. There’s a major flaw with how we can evaluate our data ….We’re coming up with a new data management system over the next year to mitigate the issue … that way, we can have better metrics and better querying of the data to actually understand the issue at large’

(#16-US, public health agency, local).

Our interviewees also spoke about the practical benefits of using qualitative approaches, such as enriching their understanding of how policies affect the community in ways that affect their outreach strategies, in their own use of data (#32-US, public health agency; #24-Canada, research, state; #17-UK, non-profit, state).

Public health advocates and regulators in tobacco control are doing this work in the context of a highly resourced and mobilised tobacco industry that presents its own data, studies, and conclusions. Industry-sponsored research is seen as a major challenge to data quality. Interviewees noted that industry ‘can choose their own research questions, their own paradigms, and this steers the discussion’, requiring public health stakeholders to be mindful of exactly who is generating evidence and to what purpose (#2-Netherlands, public health agency, national, #7-Canada, non-profit, national). Survey data are sometimes ‘so dirty and messy’ in the face of industry evasion of regulations through rapid product changes, that mixing data is sometimes the only option to understand what is happening in meaningful ways (#17-UK, non-profit, state). Retail non-compliance with regulations can also lead to data challenges, such as in situations where cash sales of flavoured products occur but are not captured in official data, or where tobacco product sales are incorrectly reported as sales of ‘miscellaneous’ products (#16-US, public health agency, local).

Discrepancies between data sources can exacerbate quality issues. It is common to find different definitions of who is a ‘smoker’ or ‘vaper,’ or who counts as a ‘youth.’ These definitions vary within and across jurisdictions, as well as over time. In one jurisdiction, eg., ‘there’s an increasing differentiation between what [government] surveys are finding and what [academic] surveys are finding in terms of smoking prevalence’, attributed to changes in how ‘smoking’ is defined in these surveys (#34-UK, non-profit, state).

Given these data limitations, some interviewees were cautious about promoting certain claims to policymakers. In the context of building policy narratives, actors warned that people should be very careful not to make generalised claims from data sources that pertain to a specific geographic or demographic population (#20-Canada, non-profit, national). Evaluation of others’ claims was seen as important due to the high political conflicts around tobacco control policies:

‘Facts should be facts. They shouldn’t be opinions being marketed as facts. I believe that because there are strong influences from tobacco lobbies, in advocacy groups, that the science should be as clean and clear as possible. And that there should always be mechanisms in place to review the science, not just on its message, but also on the quality and the strength of the evidence. I think that’s probably lacking in some policy development discussions in [my jurisdiction]’

(#24-Canada, research, state).

### 4.5. Relevance

Lastly, interviewees reported that mixing data can help support the relevance of tobacco control research and analysis to those affected by tobacco products. Tobacco control research is applied research by definition; interviewees felt that analysis should be relevant in the face of changing policy and market environments, and that the lessons that we learn from tobacco policy evaluations should be relevant to the public and, especially, to the communities most impacted by tobacco use. Approaches such as interviews, focus groups, oral history, and narrative construction were used to improve the relevance of assessments by improving understanding of the policy environment, evolving markets, community voice, and the perspectives of underrepresented groups.

Our interviews suggest that stakeholders in tobacco control consider qualitative data collection to be important in capturing community perspectives (#25-US, public health agency, state). Our interviewees, especially those involved in programme evaluation and tracking policy effects, were aware and concerned about the representativeness of data and the voice of communities impacted by tobacco and nicotine products:

‘We are trying to engage more with people with lived experience. So what we mean by that is that people who are currently or formerly use tobacco products or people who are currently using vaping products as a way to stay off of tobacco. [We want] to really hear from those with that experience, to have a better understanding of how our policies and regulations could impact them ….And with that we’ve been able to have informal conversations that have been really useful because it’s informed us [in the government]. We’ve also had two occasions now where [community members] have been able to meet with [elected health officials] to provide their perspectives’

(#39-Canada, public health agency, national).

“[We use] qualitative data from the people on the field, such as’ Does this community have strong or weak political will? Do they actually want to do tobacco control or not? All that data comes together to help us identify what are the communities where we should be doing work rather than starting from the wealthier communities’

(#26-US, public health agency, local).

Interviewees described how community partnerships influenced the design of evaluations and generation of evidence. Youth engagement and narrative building, together with findings from existing literature, often had a role alongside quantitative data on factors like use and cessation rates or uptake of monetary incentives to quit and how these differed across different populations (#32-US, public health agency, state). Sometimes these community collaborations were more expansive, moving beyond the boundaries of the tobacco control domain. Retail observations conducted by young people recruited from local communities began in tobacco control, but ‘at a certain point, we decided to collaborate with nutrition and alcohol programmes. And then, later on, the sexually transmitted disease programme joined in’ (#27-US, public health agency, state).

Finally, interviewees tied this important qualitative, community-led data collection back into the construction of policy narratives, stressing the value of voice in emphasising why policy change was needed. Mixing qualitative data with existing quantitative evaluations and surveys was seen as an important part of building narratives for policymakers:

‘Some people need to have a better understanding as to what people are thinking and feeling because that is also what impacts the work. And that’s what really motivates the change. And I think that also helps to get the youth more involved in the work when they know how someone’s life is affected by it’

(#12-US, public health agency, local).

‘Our [local community-led groups] do both secondary and primary research … We have population level data. But oftentimes those population level data, not much was actually sampled in [our jurisdiction], right?’ Because of these limitations local groups have administered targeted surveys and conducted key informant interviews, focus groups, structured observations, and photo voice projects. The qualitative data ‘was always helpful paired with something else quantitative. [interviewee holds up two hands to illustrate different data types, before bringing them tightly together] Gotcha’

(#5-US, public health agency, local).

In mixing data for policymaking and evaluation doing this work, tobacco policy stakeholders face significant challenges. Despite their express interest in accessing and using qualitative data, interviewees recognised that it can be difficult to obtain (#35, #36, #21, #9, #34). The collection and integration of qualitative data to inform tobacco control is resource intensive, and many of our interviewees work in contexts where collection and analysis needs far outweigh available resources and capacity. Sometimes the timeline for making decisions is not a good fit with available evidence or the resources to synthesise it. Some data collection is viable but does not happen due to a lack of time and or labour to complete the work (#36).

Interviewees also noted that tensions between ‘academic’ research timelines, priorities, and career advancement can be at odds with more immediate needs for applied data in policy contexts. For instance, ‘there’s something in the culture, a tobacco control culture, that doesn’t maximise the data that is available, and that prefers new data collection over data liberation’ (#20). Many tobacco control policy actors have roles that overlap with, or push up against, academic and research spaces, and some are researchers themselves in diverse ways. But in policy spaces they are performing a different vital role as translators of research and explainers of data. The differential expectations of academic and policy systems can be challenging for those whose goal is data synthesis and simplification.

### 4.6. Discussion

Our interviews make clear the value of and need for integration across different types of data in the evaluation and improvement of tobacco control regulations. Mixing data can generate information about the granularity and validity of data collected about public health, and qualitative approaches in public health are used to analyse and manage data quality (Bernardi et al., 2023). The tobacco control officials and experts we interviewed are making policy in different legal contexts, yet almost all of them talked about the important ways with which they integrate different types of qualitative and quantitative data as part of policy evaluation and formulation of alternatives on a regular basis.

Our study contributes to the literature on evidence-based policy in public health by providing a framework for understanding how tobacco control personnel use, mix, and analyse data from diverse sources. While prior literature on evidence-based policy has demonstrated the key role of public health actors and regulators in generating and translating evidence, we identify five specific ways in which personnel integrate data to inform the policy process. By interviewing personnel from six different jurisdictions at both the national and subnational level, we show how the practice of data integration is not specific to a particular jurisdiction or regulatory environment. Rather, data integration is a valuable skill that tobacco control stakeholders exercise across multiple policymaking contexts. The tobacco control case also provides insights into how data integration occurs in an environment characterised by relatively high uncertainty, conflicts with well-resourced industry players, and frequent market change.

There are several limitations to our study. We do not use an explicitly comparative approach in our analysis, though we do explore comparisons across our case studies in other related research (Ma et al., 2026). Most of our interviewees work in the U.S., since our case selection criteria required relevance of the cases for US policymakers. Nonetheless, our sample size may be subject to selection bias in our response rate and reasons for non-participation. Although we had identified and reached out to 75 individuals, we successfully interviewed slightly more than half of our initial targeted interviewee pool. The individuals who declined to participate in the study either did not respond to our initial request, could not secure permissions to schedule an interview in the requisite time frame, or were unable to participate due to budgetary or policy constraints. Lastly, qualitative research involving semi-structured interviews and in-depth case studies can provide ‘thick’ descriptions of our phenomena of interest, but statistical generalisability is inherently limited by this research design (Sartori, 1970).

Our study has several implications for policy and practice. Public health actors everywhere, especially but not only within the US, are facing significant funding challenges while making policies in the context of better-funded industry actors. This suggests that we need both better recognition of the importance of public health officials as interpreters and integrators of specialist data, as well as more and better-structured coordination between qualitative and community researchers and public health officials at all levels. This is needed in order to produce, share, and reuse useful qualitative data for both researchers and policymakers that sits alongside existing quantitative data and analysis. Qualitative work in relation to the implementation of tobacco control policies is being conducted in a piecemeal fashion by teams of researchers. This work is important but requires better coordination to prevent burdensome duplication of efforts, especially in interviews with busy public health staff working in the field.

In light of our findings, we make three specific recommendations for tobacco control policy and practice. First, public health funding is critical to support the data integration efforts of public health personnel, researchers, and regulators. As our study demonstrates, the data integration process is not easily automated or replaced; successful data integration in tobacco control relies on significant personnel and technical resources to support on-the-ground observations and persistent efforts to collect data that is often difficult to obtain. Cuts to public health pose significant threats to the staffing and efforts required to obtain, integrate, and understand the data needed for evidence-based tobacco control policy. Second, public health agencies should prioritise retention of staff and expertise, as well as foster knowledge-sharing relationships in the tobacco control epistemic network. Prior work in tobacco control knowledge management has emphasised the need to build capacity and make use of experts’ tacit knowledge in global tobacco control networks (Bickford and Kothari, 2008; Russo et al., 2017; Stillman et al., 2005). Our study demonstrates the value of tobacco regulatory science and related research infrastructure, including the FDA’s Tobacco Centers for Regulatory Science (TCORS), that encourage pluralistic, integrative approaches with clear channels for policy relevance and application (Wipfli et al., 2017). Third, tobacco policy stakeholders must communicate the importance of retaining and maintaining key data sources, such as the National Youth Tobacco Survey (NYTS) and the Population Assessment of Tobacco and Health (PATH), in concert with qualitative data-generating efforts. Established and consistent data infrastructure provides a stable and reliable source of longitudinal information that can be integrated with other data sources to track policy implementation and inform iterative change.

### 4.7. Conclusion

Evaluating tobacco control policy can be challenging, given the rapidly evolving nature of the tobacco and nicotine product market and variation in policy contexts. In the context of funding cuts and political pressure to automate policy decision-making through application of new technologies, tobacco policy stakeholders serve a core function in maintaining governance capacity. We find five distinct ways in which public health advocates and regulators who formulate, advise, and implement tobacco control policies integrate data to evaluate those policies and structure future policy decisions. Specifically, data integration performed by these actors informs assumptions, temporality, comparison, data quality, and representativeness in tobacco policy evaluation. Performing data integration requires specialisation through the development and application of specific knowledge and skills, and these efforts contribute to both the accuracy of policy evaluations and the success in achieving policy goals. Without these actors and the data integration they provide, by implication, policies would much more often miss their mark. This is not only the case in tobacco control – contemporary threats to public health funding, human capital, and infrastructure risk undermining the success of evidence-based policies in public health across a wide range of issues.

### 4.8. Policy points

- Tobacco policy stakeholders, by which we mean public health advocates working in tobacco control and tobacco control regulators, play a critical role in tobacco control efforts by integrating disparate data and sources to construct evidence and evaluate policy.
- Tobacco policy stakeholders integrate data to inform tobacco control in five specific ways: to clarify assumptions, track stages of implementation and enforcement, understand variation and comparisons across different settings, assess data quality and limitations, and explain the relevance of policy research and analysis to affected communities.
- Budget cuts and staffing reductions threaten the institutional capacity of tobacco control, as data integration by tobacco policy stakeholders is not easy to replicate or replace.

## Supplementary Material

https://doi.org/10.1017/S1744133126100619.sm003

https://doi.org/10.1017/S1744133126100619.sm002

https://doi.org/10.1017/S1744133126100619.sm001

**Supplementary material.** The supplementary material for this article can be found at https://doi.org/10.1017/S1744133126100619.

## Figures and Tables

**Figure 1.**
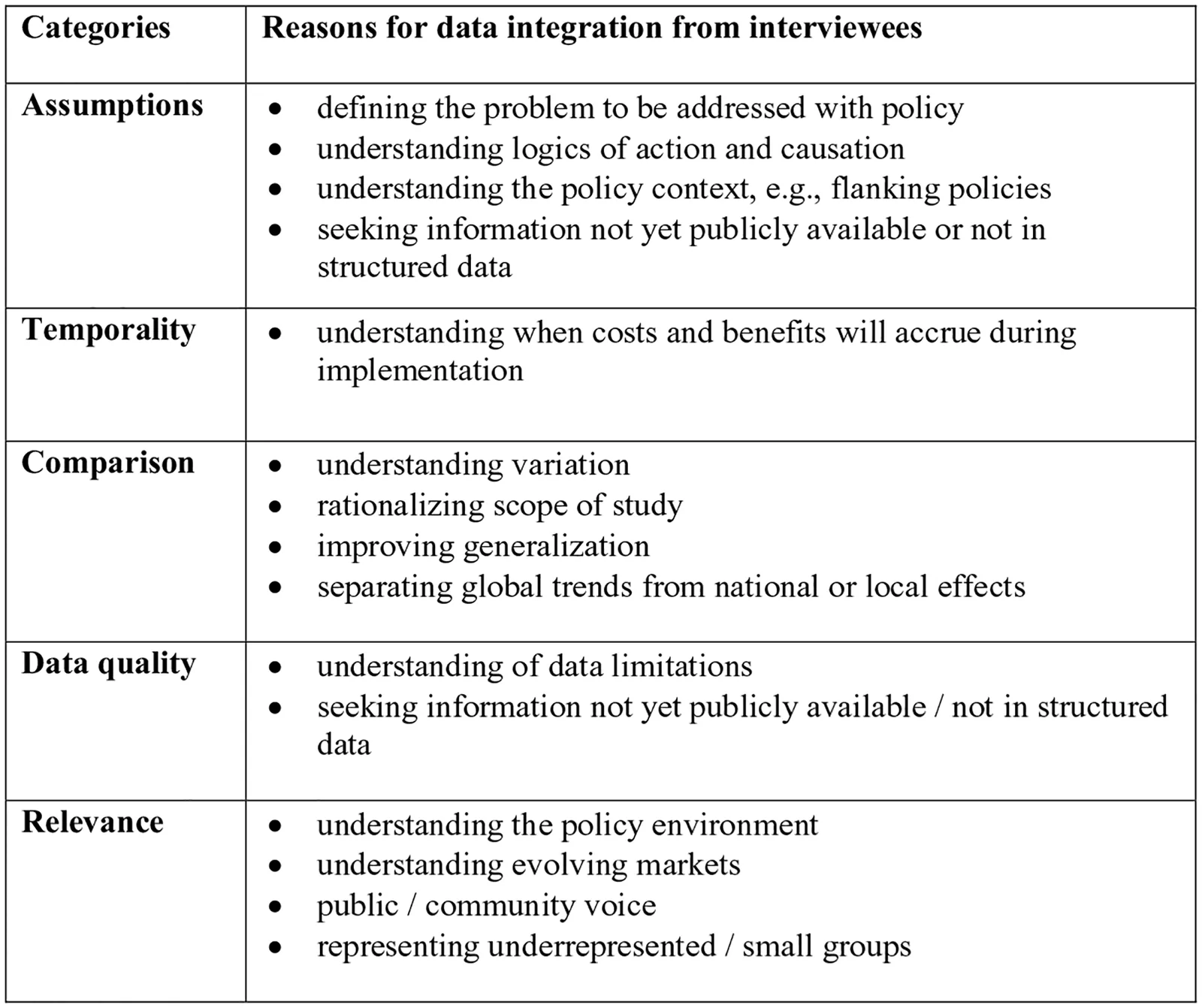
Five ways data integration informs tobacco control policy evaluation.

**Table 1.** Summary of cases, regulatory context, and interviewees

Case	Summary of regulatory context	Type of organisations where our interviewees work	Levels of government where our interviewees work	# of interviewees (total *n* =40)
US	U.S. Federal Family Smoking Prevention and Tobacco Control Act (2009) prohibits flavoured cigarettes, with the exception of menthol. FDA requires that e-cigarettes undergo pre-market authorisation. State laws vary. Our 3 U.S. cases all have more stringent prohibitions on ‘characterising flavours’ in tobacco products and/or e-cigarettes than Federal law.	Public health agency; Non-profit; Research	National; State; Local (county, city)	23
Canada	Tobacco and Vaping Products Act (amended 2024) prohibits flavouring additives and provides a permitted list of additives. Bans flavour additives and menthol in tobacco. Federal restrictions on e-cigarette flavours to only allow tobacco, mint, or menthol have not been implemented (as of March 2026). Youth-oriented flavour marketing is prohibited. Some provinces have more stringent regulations on flavoured e-cigarettes.	Public health agency; Non-profit; Research	National; State (provincial)	6
UK	EU’s Tobacco Products Directive (2014) requires that manufacturers report ingredients. UK’s Tobacco and Related Products Regulations (2016) bans ‘characterising flavours’ and menthol/cooling in cigarettes. No ban on flavours in e-cigarettes (as of March 2026, a proposed ban is under consideration).	Public health agency; Non-profit; Research	State (country); Local (city)	6
Netherlands	EU’s Tobacco Products Directive (2014) requires that manufacturers report ingredients. The Netherlands’ Tobacco and Smoking Products Act (1988, amended) allows for a permitted list of additives. Bans any ‘flavour-determining additives’ for tobacco and e-cigarettes, including components.	Public health agency; Research	National	5

**Table 2.** Illustrative quotes from interviews for the 5 categories of data integration

Categories of data integration	Examples from interviews
**Assumptions** made by policymakers/public about tobacco policies, goals, and products.	‘[Tobacco policies are] part of a broader objective. Pulling out the effects of one policy, like ‘if you do X, it will have Y impact’… there are only a very few policies [for which] you can do that’ (#34-UK, non-profit, state).
**Temporality** i.e., timing of policy implementation and enforcement.	‘We have adult tobacco use data from [Year 1] and [Year 0]. And our policy went into effect at the beginning of [Year 1]. So we’ve had researchers contacting us, wanting to compare those two years. But the problem is the data is collected in early [Year 1]… it wasn’t like you flipped a switch, and the law was implemented’ (#27-US, public health agency, state). ‘Even if we could have gotten all the notifications [about the new flavour law] sent to retailers right then, it probably wouldn’t have been a good time for them to be getting mail … we sent out the mailings [after the holiday period], and it takes a long time to send postcards to [thousands of] retailers … They were even just getting the official mailing, notifying them about the law throughout [the month after it was in effect]’ (#27-US, public health agency, state).
**Comparison** between cases, jurisdictions, and settings.	‘In [other jurisdictions], major groups in political life support a package of tobacco control measures. That’s not the case [in our jurisdiction]. I would say that [other jurisdictions are] better placed for such a measure than [our jurisdiction] right now’ (#30-Netherlands, public health agency, national).
**Data Quality** i.e., information about the completeness and structure of the data.	‘I think qualitative data can be really powerful. And reaching the folks that are most directly impacted by the policy and have been addicted to menthol [products] and are experiencing barriers to cessation. And sometimes we don’t necessarily reach those folks in our normal data sources. We’ve tried to come up with different strategies to increase representation in data collection - we have an online panel survey that’s a little bit different from our traditional phone surveillance surveys. Our vendor sends out direct household mailings which has helped reach folks we don’t normally reach in data collection’ (#32-US, public health agency). ‘I think we need to do qualitative work on the field and make sure that we are capturing minoritised or vulnerable groups that tend to be over-impacted by the use of vaping products to understand not only population trends, but also some subgroups of young people’ (#24-Canada, research, state). ‘To circumvent lots and lots of questions [for a survey], a number of qualitative interviews … allows you to get much richer data than you can ever get from a survey’ (#17-UK, non-profit, state).
**Relevance** of the research for the policy context, market, and communities.	‘We’ve been providing some training to our grantees to have them doing some neighbourhood conversations and getting some community local input … [and] to get a pulse of what’s going on in the community, too. So that’s something we’re trying to infuse regularly’ (#25-US, public health agency, state).

## Footnotes

1 While our study examines the production and use of evidence by tobacco control personnel, we do not attempt to make an overall evaluation of the effect of these tobacco control policies; there exists significant policy variation among jurisdictions, and assessment of causal effects of tobacco policies is ongoing.

2 Terminology used to describe this type of research includes Qualitative and Multi-Method Research (‘QMMR’), Mixed Methods Research, and Qualitatively-oriented Mixed Methods Research. Each approach differs slightly, but data integration is common to each.

3 Created by adding ingredients such as menthol or clove.

4 The WHO refers to these products as novel and emerging nicotine and tobacco products (NENTPs).

5 Countries that have partial or comprehensive flavour restrictions include the US, Brazil, Canada, Ethiopia, EU (28 member states), Uganda, Senegal, Niger, Mauritania, Moldova, Turkey and Singapore (Erinoso et al., 2021). The Campaign for Tobacco-Free Kids maintains an ongoing database of current tobacco laws worldwide, including flavour restrictions. In recent years, more countries have chosen to restrict flavours in tobacco products using different mechanisms and definitions.

6 Interviewee numbers are randomised so the numbers do not denote the order in which interviews were conducted.

7 The GDPR can be found here: https://gdpr.eu/.

8 Interview quotes have been paraphrased for clarity. Location and date information has been changed in order to protect the anonymity of interviewees.

## References

[R1] Andersen NA and Breidahl KN (2021) The power of ideas in policymaking processes: The role of institutionalised knowledge production in state bureaucracies. Social Policy & Administration 55(5), 848–862. 10.1111/spol.12665

[R2] Audrain-McGovern J, Manikandan D, Koita F, Klapec O, Pickworth WB and Stone MD (2025) Effect of sweet flavouring on the rewarding and reinforcing value of cigarillo use among young adults. Tobacco Control 34(3), 294–301. 10.1136/tc-2023-058307 PMC11116272 38050181

[R3] Baron J (2018) A brief history of evidence-based policy. The ANNALS of the American Academy of Political and Social Science 678(1), 40–50. 10.1177/0002716218763128

[R4] Béland D (2005) Ideas and social policy: An institutionalist perspective. Social Policy & Administration 39(1), 1–18. 10.1111/j.1467-9515.2005.00421.x

[R5] Béland D (2009) Ideas, institutions, and policy change. Journal of European Public Policy 16(5), 701–718. 10.1080/13501760902983382

[R6] Bernardi FA, Alves D, Crepaldi N, Yamada DB, Lima Vícius C and Rijo R (2023) Data quality in health research: Integrative literature review. Journal of Medical Internet Research 25(1), e41446. 10.2196/41446 PMC10646672 37906223

[R7] Bickford JJ and Kothari AR (2008) Research and knowledge in Ontario tobacco control networks. Canadian Journal of Public Health 99(4), 297–300. 10.1007/BF03403759 PMC6975625 18767275

[R8] Brownson RC, Chriqui JF and Stamatakis KA (2009) Understanding evidence-based public health policy. American Journal of Public Health 99(9), 1576–1583. 10.2105/AJPH.2008.156224 PMC2724448 19608941

[R9] Büthe T, Jacobs A, Bleich E, Pekkanen R, Trachtenberg M, Cramer K, Shih V, Parkinson S, Pachirat T, Romney D, Stewart BM, Tingley D, Davison A, Schneider C, Wagemann C and Fairfield T (2015) Transparency in qualitative and multi-method research: A symposium. Qualitative and Multi-Method Research: Newsletter of the American Political Science Association’s QMMR Section 13(1), 2–64.

[R10] Cairney P and Oliver K (2017) Evidence-based policymaking is not like evidence-based medicine, so how far should you Go to bridge the divide between evidence and policy? Health Research Policy and Systems 15(1), 35. 10.1186/s12961-017-0192-x PMC5407004 28446185

[R11] Cairney P, Studlar DT and Mamudu HM (2012) Global Tobacco Control. Palgrave Macmillan UK. 10.1057/9780230361249

[R12] Campaign for Tobacco-Free Kids (2026) ‘Tobacco Control Laws.’ Tobacco Control Laws. Available at https://www.tobaccocontrollaws.org/legislation/find-by-policy

[R13] CDC (2024a) ‘Burden of Tobacco Use in the U.S.’ Government. Centers for Disease Control and Prevention, Centers for Disease Control and Prevention, October 8. Available at https://www.cdc.gov/tobacco/campaign/tips/resources/data/cigarette-smoking-in-united-states.html (accessed 13 March 2026).

[R14] CDC (2024b) Economic Trends in Tobacco. Smoking and Tobacco Use, September 13. Available at https://www.cdc.gov/tobacco/php/data-statistics/economic-trends/index.html (accessed 28 May 2025).

[R15] Chen H, Hailey D, Wang N and Yu P (2014) A review of data quality assessment methods for public health information systems. International Journal of Environmental Research & Public Health 11(5), 5. 10.3390/ijerph110505170 PMC4053886 24830450

[R16] Clavier C, Gagnon F and Poland B (2022) Sidestepping the stalemate: The strategies of public health actors for circulating evidence into the policy process. In Fafard P Cassola A and de Leeuw E (ed.), Integrating Science and Politics for Public Health. Springer Nature. https://directory.doabooks.org/handle/20.500.12854/84353.

[R17] Cohen G and Cook S (2025) Observational studies of exposure to tobacco and nicotine products: Best practices for maximizing statistical precision and accuracy. iScience 28(3), 111985 10.1016/j.isci.2025.111985 PMC11915159 40104063

[R18] Cohen JE, Amalia B, Luo W, McWhirter KJ, Masanga BC and Pankow JF (2024) Eugenol, menthol and other flavour chemicals in Kreteks and ‘White’ cigarettes purchased in Indonesia. Original Research. Tobacco Control 33(5), 637–640. 10.1136/tc-2022-057827 PMC11503103 37094935

[R19] Cohen JE, Welding K, Erinoso O, Saraf S, Iacobelli M and Smith KC (2021) The flavor train: The nature and extent of flavored cigarettes in low-and middle-income countries. Nicotine & Tobacco Research 23(11), 1936–1941. 10.1093/ntr/ntab092 PMC8496476 33983445

[R20] Creswell J, Klassen AC, Plano V and Smith KC (2011) Best Practices for Mixed Methods Research in the Health Sciences. National Institutes of Health.

[R21] da Silva ALO, Bialous SA, Albertassi PGD, dos Reis Arquete DA, Fernandes AMMS and Moreira JC (2019) The taste of smoke: tobacco industry strategies to prevent the prohibition of additives in tobacco products in Brazil. Tobacco Control 28(e2), e92–101. 10.1136/tobaccocontrol-2018-054892 31152115

[R22] Derthick MA (2011) Up in Smoke: From Legislation to Litigation in Tobacco Politics, 3rd Edn. Washington, D.C.: CQ Press.

[R23] Erinoso O, Smith KC, Iacobelli M, Saraf S, Welding K and Cohen JE (2021) Global review of tobacco product flavour policies. Original Research. Tobacco Control 30(4), 373–379. 10.1136/tobaccocontrol-2019-055454 PMC8237183 32414867

[R24] Fafard P Cassola A and de Leeuw E (eds.) (2022) Integrating Science and Politics for Public Health. Springer Nature. https://directory.doabooks.org/handle/20.500.12854/84353

[R25] FDA (2025) DA Launches Agency-Wide AI Tool to Optimize Performance for the American People.’ FDA, FDA, June 3. https://www.fda.gov/news-events/press-announcements/fda-launches-agency-wide-ai-tool-optimize-performance-american-people

[R26] Fielding NG (2012) Triangulation and mixed methods designs: Data integration with new research technologies. Journal of Mixed Methods Research 6(2), 124–136. 10.1177/1558689812437101

[R27] Fooks G, Gilmore A, Collin J, Holden C and Lee K (2013) The limits of corporate social responsibility: Techniques of neutralization, stakeholder management and political CSR. Journal of Business Ethics 112(2), 283–299. 10.1007/s10551-012-1250-5 PMC3755635 23997379

[R28] Gerring J (2007) Case Study Research: Principles and Practices. New York, NY: Cambridge University Press.

[R29] Gilmore AB, Fabbri A, Baum F, Bertscher A, Bondy K, Chang H-J, Demaio S, Erzse A, Freudenberg N, Friel S, Hofman KJ, Johns P, Abdool Karim S, Lacy-Nichols J, de Carvalho CMP, Marten R, McKee M, Petticrew M, Robertson L, Tangcharoensathien V, Thow AM (2023) Defining and conceptualising the commercial determinants of health. Lancet (London, England) 401(10383), 1194–1213. 10.1016/S0140-6736(23)00013-2 36966782

[R30] Gupta K (2012) Comparative public policy: Using the comparative method to advance our understanding of the policy process. Policy Studies Journal 40(s1), 11–26. 10.1111/j.1541-0072.2012.00443.x

[R31] Head BW (2016) Toward more ‘Evidence-informed’ policy making? Public Administration Review 76(3), 472–484. 10.1111/puar.12475

[R32] Hiscock R, Alaouie H, Matthes BK, Mehegan J and Bloomfield MJ (2023) Hosting the tobacco industry supply chain and political interference. Nicotine and Tobacco Research 25(12), 1847–1855. 10.1093/ntr/ntad178 PMC10664082 37707978

[R33] Hiscock R, Silver K, Zatoński M and Gilmore AB (2020) Tobacco industry tactics to circumvent and undermine the menthol cigarette ban in the UK. Industry watch. Tobacco Control 29(e1), e138–42. 10.1136/tobaccocontrol-2020-055769 PMC7799418 32418921

[R34] Jacobson PD and Wasserman J (1999) The implementation and enforcement of tobacco control laws: Policy implications for activists and the industry. Journal of Health Politics, Policy and Law 24(3), 567–598. 10.1215/03616878-24-3-567 10386327

[R35] Kickbusch I (2012) Addressing the interface of the political and commercial determinants of health. Health Promotion International 27(4), 427–428. 10.1093/heapro/das057 23144264

[R36] Kroneman MW and van der Zee J (1997) Health policy as a fuzzy concept: methodological problems encountered when evaluating health policy reforms in an international perspective. Health Policy 40(2), 139–155. 10.1016/S0168-8510(96)00890-1 10167068

[R37] Leventhal AM, Mason TB, Cwalina SN, Whitted L, Anderson M and Callahan C (2020) Flavor and nicotine effects on E-cigarette appeal in young adults: Moderation by reason for vaping. American Journal of Health Behavior 44(5), 732–743. 10.5993/AJHB.44.5.15 PMC8284482 33121589

[R38] Levy DT, Liber AC, Cadham C, Sanchez-Romero LM, Hyland A, Cummings M, Douglas C, Meza R, Henriksen L (2023) Follow the money: A closer look at US tobacco industry marketing expenditures. Tobacco Control 32(5), 575–582. 10.1136/tobaccocontrol-2021-056971 PMC9346571 35074930

[R39] Lindson N, Livingstone-Banks J, Butler AR, Levy DT, Barnett P, Theodoulou A, Notley C, Rigotti NA, Chen Y, Hartmann-Boyce J (2025) An update of a systematic review and meta-analyses exploring flavours in intervention studies of e-cigarettes for smoking cessation. Addiction (Abingdon, England) 120(4), 770–778. 10.1111/add.16736 PMC11907327 39702981

[R40] Ling PM, Kim M, Egbe CO, Patanavanich R, Pinho M and Hendlin Y (2022) Moving targets: how the rapidly changing tobacco and nicotine landscape creates advertising and promotion policy challenges. Special Communication. Tobacco Control 31(2), 222–228. 10.1136/tobaccocontrol-2021-056552 PMC9233523 35241592

[R41] Livingstone-Banks J, Travis N, Conde M, Chen Y (Crystal), Zi P, Jarman H, Lindson N, Hartmann-Boyce J (2025) The impacts of E-cigarette flavours: an overview of systematic reviews. Addiction 120(7), 1327–1344. 10.1111/add.70017 PMC12128567 39999998

[R42] Ma C, Jarman H, Vissapragada P and Hartmann-Boyce J (2026) 3–129. A Global Comparison of Flavor Restrictions for Cigarettes and E-Cigarettes.’ Paper presented at SRNT 2026, Baltimore, MD. SRNT 2026, March 4.. Available at https://srnt.org/wp-content/uploads/2026/06/SRNT-2026-Abstract-Book.pdf

[R43] McNeill A, Simonavičius E, Brose L, Taylor E, East K, Zuikova E, Calder R, Robson D (2022) Nicotine vaping in England: an evidence update including health risks and perceptions. Office for Health Improvement and Disparities. https://www.gov.uk/government/publications/nicotine-vaping-in-england-2022-evidence-update (accessed 15 January 2026).

[R44] Mertens DM (2016) Assumptions at the philosophical and programmatic levels in evaluation. Evaluation and Program Planning 59(December), 102–108. 10.1016/j.evalprogplan.2016.05.010 27340047

[R45] Mosley L (ed.) (2013) Interview Research in Political Science. 1 edition. Cornell University Press.

[R46] National Academies of Sciences, Engineering, and Medicine (NASEM) (2018) Public Health Consequences of E-Cigarettes. National Academies of Sciences, Engineering, and Medicine. Available at https://www-nap-edu.proxy.lib.umich.edu/resource/24952/012318ecigaretteHighlights.pdf

[R47] Peck K, Rodericks R, Irvin L, Johnson L, Tamashiro J, Ching L, Sentell T, Pirkle C (2022) Identifying best practices in adoption, implementation and enforcement of flavoured tobacco product restrictions and bans: Lessons from experts. Tobacco Control 31(1), 32–39. 10.1136/tobaccocontrol-2020-055884 33067409

[R48] Poth CN, Wongvorachan T, Bulut O and Otto SJG (2024) Adaptive case study-mixed methods design practices for researchers studying complex phenomena. Journal of Mixed Methods Research 18(3), 292–303. 10.1177/15586898241250217

[R49] Prinja S (2010) Role of ideas and ideologies in evidence-based health policy. Iranian Journal of Public Health 39(1), 64–69. PMC3468969 23112991

[R50] Pullicin AJ, Kim H, Brinkman MC, Buehler SS, Clark PI and Lim J (2020) Impacts of nicotine and flavoring on the sensory perception of E-cigarette aerosol. Nicotine & Tobacco Research 22(5), 806–813. 10.1093/ntr/ntz058 PMC7171271 30997500

[R51] Ragin CC, Berg-Schlosser D and de M (1998) Political methodology: Qualitative methods. In Robert Goodin G and Klingemann H-D (eds.)Oxford University Press. https://global.oup.com/academic/product/a-new-handbook-of-political-science-9780198294719?cc=us&lang=en&

[R52] Ramanathan T, Hulkower R, Holbrook J and Penn M (2017) Legal epidemiology: The science of law. The Journal of Law, Medicine & Ethics 45(1_suppl), 69–72. 10.1177/1073110517703329 PMC5690565 28661299

[R53] Russo AR, Solis AC, Villanti AC, Wipfli HL, Kern TT, Lawley RK, Collins LK, Abudayyeh HS, Chansky MC, Glantz SA, Samet JM, Benjamin EJ (2017) Mentoring for success in tobacco regulatory science: a qualitative study. Tobacco Regulatory Science 3(3), 280–292. 10.18001/TRS.3.3.4 PMC5533280 28758143

[R54] Saldaña J (2015) The Coding Manual for Qualitative Researchers, 3rd Edn. Thousand Oaks, CA: SAGE Publications, Inc.

[R55] Sartori G (1970) Concept misformation in comparative politics. American Political Science Review 64(04), 1033–1053. 10.2307/1958356

[R56] Skivington K, Matthews L and Simpson SA, Craig P, Baird J, Blazeby JM, Boyd KA, Craig N, French DP, McIntosh E, Petticrew M, Rycroft-Malone J, White M, Moore L (2021) A new framework for developing and evaluating complex interventions: update of medical research council guidance. Research Methods & Reporting. BMJ 374(September), n206 1. 10.1136/bmj.n2061 PMC8482308 34593508

[R57] Spector AY and Pinto RM (2017) Partnership matters in health services research: A mixed methods study of practitioners’ involvement in research and subsequent use of evidence-based interventions. Journal of Mixed Methods Research 11(3), 374–393. 10.1177/1558689815619823

[R58] Stillman FA, Wipfli HL, Lando HA, Leischow S and Samet JM (2005) Building capacity for international tobacco control research: The global tobacco research network. American Journal of Public Health 95(6), 965–968. 10.2105/AJPH.2004.047183 PMC1449292 15914817

[R59] Talhout R and Leventhal AM (2026) Coolants, organic acids, flavourings and other additives that facilitate inhalation of tobacco and nicotine products: Implications for regulation. Tobacco Control 35, 110–116. 10.1136/tc-2024-058738 PMC12911559 39256038

[R60] Travis N, Knoll M, Cadham CJ, Cook S, Warner KE, Fleischer NL, Douglas CE, Sánchez-Romero LM, Mistry R, Meza R, Hirschtick JL, Levy DT (2022) Health effects of electronic cigarettes: An umbrella review and methodological considerations. International Journal of Environmental Research & Public Health 19(15), 9054. 10.3390/ijerph19159054 PMC9330875 35897421

[R61] Travis N, Warner KE, Goniewicz ML, Oh H, Ranganathan R, Meza R, Hartmann-Boyce J, Levy DT (2025) The potential impact of oral nicotine pouches on public health: A scoping review. Nicotine & Tobacco Research 27(4), 598–610. 10.1093/ntr/ntae131 PMC11931220 38880491

[R62] Whitacre TR, Crippen A, Monthrope M, Narine T, Liber AC and Friedman AS (2025) Tobacco product flavour policies in the USA. Tobacco Control 34, 688–693. 10.1136/tc-2024-058895 PMC12501946 39870520

[R63] WHO (2025) Tobacco. International Organization. World Health Organization, WHO. https://www.who.int/news-room/fact-sheets/detail/tobacco.

[R64] Wickham RJ (2020) The biological impact of menthol on tobacco dependence. Nicotine & Tobacco Research 22(10), 1676–1684. 10.1093/ntr/ntz239 31867627

[R65] Wipfli HL, Berman M, Hanson K, Kelder S, Solis A, Villanti AC, Ribeiro CMP, Meissner HI, Anderson R (2017) Defining tobacco regulatory science competencies. Nicotine & Tobacco Research: Official Journal of the Society for Research on Nicotine and Tobacco 19(2), 222–230. 10.1093/ntr/ntw178 PMC5234367 27613917

