# Supplementary material for: Policy actors as data integrators: mixing data in tobacco control evaluation and assessment
Source: Health Econ Policy Law. Author manuscript; Available in PMC 2026 Oct 28. (PMC13714878; doi:10.1017/S1744133126100619)
Supplement: Supplementary file 2 [file NIHMS2218060-supplement-https___doi_org_10_1017_S1744133126100619_sm001.docx]

Supplemental A

Interview protocol topics and example questions for semi-structured, open-ended interviews

| **Topic** | **Example questions** |
| --- | --- |
| Personal background | *Can you describe your current role in terms of your involvement with tobacco product / nicotine product regulation?* |
| General regulatory priorities | *In general, what do you see as the most important regulatory challenges facing your organization right now?*  *Do you think your organization finds it challenging to evaluate evidence presented by tobacco control advocates, industry or other governments; access necessary data; manage relationships with key stakeholders; manage communications with the media and the public; etc.?*  *Why do you think [specific aspects of regulation] are challenging?*  *What kind of advice do you give to [other stakeholders] who are interested in implementing flavor restrictions?* |
| Flavor restrictions | *In trying to introduce flavor restrictions, what were / are the most significant roadblocks your organization experienced?*  *What do you think are the key factors that facilitate implementation and compliance with [the current policies?]*  *In your opinion, what other policy measures have to be in place in order for flavor restrictions to be successfully implemented?*  *How did your organization / government assess the scientific evidence on flavor restrictions?*  *What kind of data and evidence did you rely on (to support your actions in regulation, to make cases in court, etc.)?* |
| Future policymaking | *What would be your advice to regulators in other jurisdictions who are considering introducing or implementing flavor restrictions?*  *What kind of data do you need?*  *Do you see a role for different types of analysis or evidence gathering? What do you wish was available?* |
