# Supplementary material for: Policy actors as data integrators: mixing data in tobacco control evaluation and assessment
Source: Health Econ Policy Law. Author manuscript; Available in PMC 2026 Oct 28. (PMC13714878; doi:10.1017/S1744133126100619)
Supplement: Supplementary file 3 [file NIHMS2218060-supplement-https___doi_org_10_1017_S1744133126100619_sm003.docx]

Supplemental C: Additional information on interview methodology and analysis

Recruitment

Interviewees were recruited via email during fall of 2024 and identified using online official public records, tobacco-specific research networks, referrals, and personal contacts.

As part of the informed consent process, we provided our potential participants with a description of our aims in conducting the research, which were reiterated at the beginning of the interview.

This initial recruitment process also included attestations by the researchers that the researchers did not have a conflict of interest with the tobacco industry.

As is typical in semi-structured elite interviewing, we did not provide participants with the interview protocol prior to the interview.

Interview protocol and analysis

To mitigate potential bias in the data collection, analysis, and interpretation process, we maintained an open-ended interview structure to allow participants to frame issues in their own terms and independently coded the interview transcripts.
